# Transmission of varicella zoster virus in the presence of masking

**DOI:** 10.1017/ash.2024.358

**Published:** 2024-09-04

**Authors:** Luke Sequeira, Lorraine Maze dit Mieusement, Heather Candon, Aikta Verma, Jerome A. Leis

**Affiliations:** 1 Sunnybrook Health Sciences Centre, Toronto, ON, Canada; 2 Public Health Sciences, Queen’s University, Kingston, ON, Canada; 3 Department of Medicine, University of Toronto, Toronto, ON, Canada; 4 Institute of Health Policy, Management and Evaluation, and Centre for Quality Improvement and Patient Safety, University of Toronto, Toronto, ON, Canada

Healthcare-associated transmission of varicella zoster virus (VZV) has become uncommon in the context of childhood vaccination and screening of healthcare workers for immunity at start of employment.^1^ Even less experience is available since the introduction of universal masking of healthcare institutions during the COVID-19 pandemic. In our province in Canada, mask mandates were implemented from 2020 to 2022 across hospitals. After these were lifted, universal masking has continued across many healthcare sectors, including among many patients who continue to prefer to don a mask.

**Figure 1.**
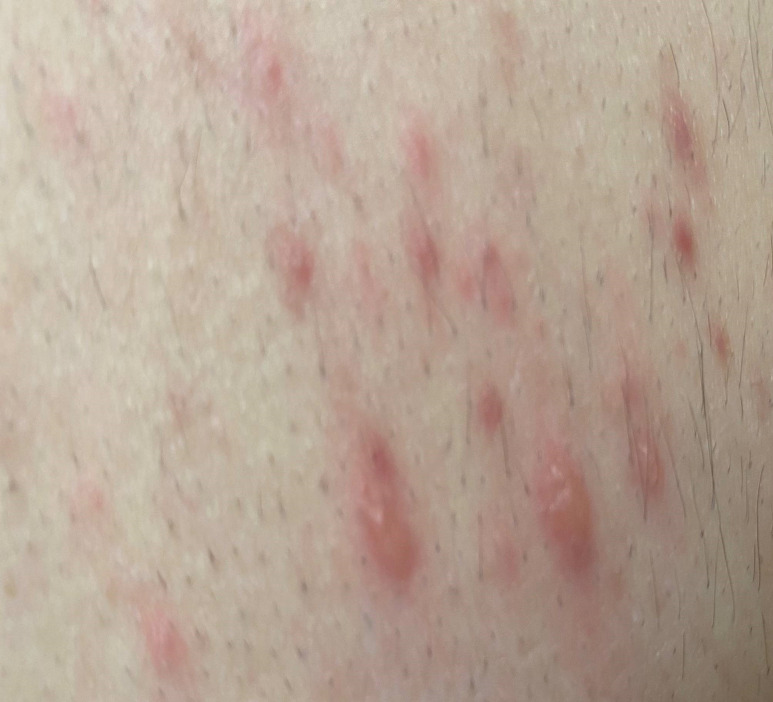
Clinical image of back of index patient revealing the typical crops of vesicular eruptions compatible with varicella zoster infection.

A man in his 30s (patient A) presented to our Emergency Department (ED) in November, 2023 with a four-day history of fever and generalized non-crusted vesicular rash. He had recently traveled, and had no history of Chickenpox or VZV vaccine. Based on the appearance of the rash (Figure 1), and virtual consultation with dermatology, the clinical diagnosis of primary VZV infection was made. The patient had spent 4 hours in the waiting room before being placed in airborne precautions. This room is 27.5 cubic meters with supply hourly air change rate of 15.2.

Contact tracing was performed for all patients who overlapped in the waiting room as per international guidelines.^2^ Contacts who remained in hospital or were discharged and considered at high risk for complications from VZV infection received follow-up to verify immunity and offer prophylaxis, as appropriate.

Sixteen days later, a man in his 40s (patient B) presented to the ED with fever and vesicular rash. Vesicular fluid was positive for VZV by polymerase chain reaction and serum VZV IgM was positive while IgG was nonreactive. On review, patient B had been identified as a contact to patient A, but immunity was never verified because he had been discharged prior and was not considered at high risk for complications, as per local public health guidelines.^3^ The patient had no other known exposures to VZV in the interim. Transmission from patient A to patient B was presumed based on the known incubation period of VZV but details surrounding the exposure were lacking.

Closed-circuit (CCTV) security camera footage was used to confirm proximity, duration, interactions, and presence of masking between patient A and patient B at time of exposure. To confirm identify of both patients in the waiting room, the registration kiosk footage was reviewed at known registration time to obtain positive identification.

CCTV during the entire exposure period revealed that patients A and B shared a cumulative total of 265 minutes in the waiting room. They sat 3.51 meters apart for 91 minutes and then patient B changed chairs and remained 2.06 meters from patient A for the remaining 174 minutes. Patient A wore a KN95 mask, hood, and long sleeves, while patient B wore a medical mask. Both patients never removed their masks, never interacted, and were never face-to-face with each other. They never had direct contact with each other or the same environmental surfaces.

To our knowledge, this is the first documented healthcare-associated transmission of VZV in the era of universal patient masking, where the source wore a KN95 and the exposed case wore a medical mask. CCTV confirmed lack of close contact, perfect adherence to masking, and that all skin lesions remained fully covered throughout exposure.

This type of transmission event is not unexpected given the known mode of transmission of VZV and may be highly communicable via inhalation of infectious respiratory particles. Previous investigations over decades have identified similar healthcare-associated transmissions of VZV, particularly across units of susceptible patient populations.^4,5^ A key difference from previous investigations is that throughout the COVID-19 pandemic, the use of masking including respirators has become more widely adopted by the general public. In this case, a KN95 respirator which was not fit-tested was used by the source patient and did not prevent transmission of VZV. This observation underscores that even in the presence of masking, VZV exposures require meticulous contact tracing of susceptible patients.

A limitation of our investigation is the lack of clinical specimen available from index patient that would have allowed for whole genome sequencing. Although we cannot fully exclude a separate VZV exposure in the community for patient B, local incidence is low and the onset of symptoms of patient B from time of ED exposure fell close to the median VZV incubation period.

The use of CCTV as a tool for public health investigations was extensively used in the COVID-19 pandemic and its application in this case was helpful in improving our understanding of this VZV transmission event beyond the use of exposure time alone. Our method for identification of affected patients by CCTV allowed us to determine the precise duration and proximity, face masks worn and how they were worn. This approach may be useful to facilitate contact tracing and risk assessment for other communicable diseases.
