# Transmission of varicella zoster virus in the presence of masking – CORRIGENDUM

**DOI:** 10.1017/ash.2024.477

**Published:** 2024-12-20

The volume of the waiting room reported in the article^1^ was not correct. While this error does not change the interpretation of the article, the authors wish to make this correction and clarify that the room measured was the waiting room where the exposure occurred.

Where the article states, “This room is 27.5 cubic meters with supply hourly air change rate of 15.2,” the article should instead read as follows: “This waiting room is 393 cubic meters with supply hourly air change rate of 15.2.”
